# circRNA Mediates Silica-Induced Macrophage Activation Via HECTD1/ZC3H12A-Dependent Ubiquitination: Erratum

**DOI:** 10.7150/thno.72553

**Published:** 2022-04-26

**Authors:** Zewei Zhou, Rong Jiang, Xiyue Yang, Huifang Guo, Shencun Fang, Yingming Zhang, Yusi Cheng, Jing Wang, Honghong Yao, Jie Chao

**Affiliations:** 1 Department of Physiology, School of Medicine, Southeast University, Nanjing, Jiangsu, 210009, China;; 2 Department of Pharmacology, School of Medicine, Southeast University, Nanjing, Jiangsu, 210009, China;; 3 Department of Respiration, Zhongda Hospital, School of Medicine, Southeast University, Nanjing, Jiangsu 210009, China;; 4 Key Laboratory of Developmental Genes and Human Disease, Southeast University, Nanjing, 210096, China;; 5 Nine Department of Respiratory Medicine, Nanjing Chest Hospital, Nanjing, Jiangsu, 210029, China;; 6 Department of Respiratory Medicine, Nanjing Drum Tower Hospital, Nanjing, Jiangsu, 210029, China;; 7 Department of Respiratory Medicine, First Affiliated Hospital of Nanjing Medical University, Nanjing, Jiangsu 210029, China.

In the original publication 1, errors were found in Fig 6B. During the assembling of figures, in Fig 6B, the gel of western blot images of GAPDH was misused. The correct figures are shown below. The authors confirm that these corrections do not change the result interpretation or conclusions of the article. The authors are deeply sorry and sincerely apologize for any inconvenience or misunderstanding that may have caused.

## Figures and Tables

**Figure 6:**
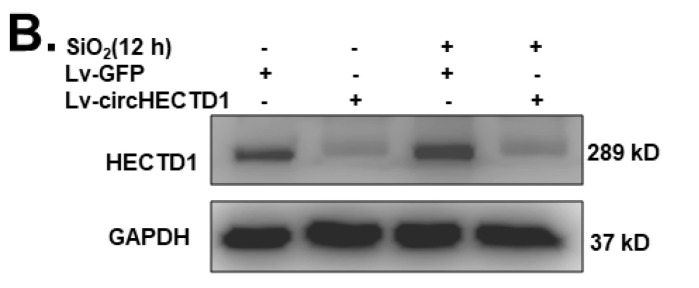
Corrected figure.
